# SProt: sphere-based protein structure similarity algorithm

**DOI:** 10.1186/1477-5956-9-S1-S20

**Published:** 2011-10-14

**Authors:** Jakub Galgonek, David Hoksza, Tomáš Skopal

**Affiliations:** 1 Siret Research Group, Faculty of Mathematics and Physics, Charles University in Prague, Malostranské nám. 25, 118 00 Prague, Czech Republic

## Abstract

**Background:**

Similarity search in protein databases is one of the most essential issues in computational proteomics. With the growing number of experimentally resolved protein structures, the focus shifted from sequences to structures. The area of structure similarity forms a big challenge since even no standard definition of optimal structure similarity exists in the field.

**Results:**

We propose a protein structure similarity measure called SProt. SProt concentrates on high-quality modeling of local similarity in the process of feature extraction. SProt’s features are based on spherical spatial neighborhood of amino acids where similarity can be well-defined. On top of the partial local similarities, global measure assessing similarity to a pair of protein structures is built. Finally, indexing is applied making the search process by an order of magnitude faster.

**Conclusions:**

The proposed method outperforms other methods in classification accuracy on SCOP superfamily and fold level, while it is at least comparable to the best existing solutions in terms of precision-recall or quality of alignment.

## Background

The biological function of a protein is consequence of its spatial conformation rather than of ordering of its amino acids (protein sequence). Thus, the protein structure is closer to the function than the sequence, therefore there was an enormous effort spent on protein structure research. Moreover, the biological motivation for protein structure similarity stems from the thesis that proteins having similar structures also share similar function. Hence, it is very useful to have tools for measuring protein structure similarity in order to be able to identify similar protein structures from a database of protein structures with already known function.

Most of the protein structure similarity measures are based on comparisons of positions of amino acids in the space. For this purpose, amino acids are represented as coordinates of their *α*-carbon (and sometimes *β*-carbon) atoms. The protein structure similarity assessment usually comprises two steps. In the first one, which we call *alignment search*, an amino acid inter-protein pairing is established. The second step, which we call *superposition search*, includes superposition optimizing the selected similarity function. This function usually aggregates values based on spatial (euclidean) distances of the paired amino acids after the superposition.

Although it has been shown that if the optimal solution is required, the above defined measuring of structure similarity is NP-hard [1] (non-deterministic polynomial-time), each step of the problem can be solved in polynomial time using the result of the other step. If we know the alignment, there exist methods how to obtain superposition optimizing given similarity formula in polynomial time, e.g., the *Kabsch* algorithm [2] for root mean square deviation (RMSD). On the other hand, if we are provided with the superposition and the similarity formula in a form of sum, we can use dynamic programming to determine the optimal alignment with respect to the given superposition. The dynamic programming has to employ a scoring corresponding to the inner part of the sum. If using RMSD, the score of *i*-th and *j-*th amino acids of the superposed proteins is defined as their squared euclidean distance. As the solution of structure similarity consists of steps that depend each on the other, at the beginning we do not know neither the alignment nor the superposition.

We briefly describe the main ideas of some of the state-of-the-art algorithms and also some solutions which outperform the other ones and which we compare to our contribution — *DALI*, *ProtDex2*, *CE*, *SSAP*, *MAMMOTH*, *Vorometric*, *Vorolign*, *PPM*, *db-iTM*, *BLAST*, *PSI-BLAST*, *3D-BLAST*. 

One of the earliest approaches to protein structure similarity assessment was *DALI*, representing the protein’s structure by a two-dimensional matrix of inter-residual distances [3,4]. Similar protein structures should also share similar distance distribution, thus in the comparison process the matrices are split into overlapping parts and similar (contact) patterns are stored. These are further extended to obtain the alignment.

Similarly to DALI, *ProtDex2* uses intra-residual distance matrices [5]. However, instead of chaining the contact patterns, ProtDex2 splits them to constant-sized submatrices which, together with their description, are used as index terms for inverted index. Protein structures used as queries are processed in the same way. The inverted index together with subsequent scoring is utilized to identify similar protein structures in the database. The query result could be furthermore refined by an arbitrary alignment-based algorithm.

The *CE* method uses the concept of aligned fragment pairs (AFP) for searching structurally similar portions of the sequences [6]. In particular, a few seeds are chosen and iteratively extended by chaining with other AFPs. Three different measures are taken into account when deciding whether a new AFP should be added to the chain. At the end, a final optimization is performed which results in the best alignment.

The *SSAP* method [7] heavily exploits Smith-Waterman dynamic programming algorithm [8]. Each residue is represented by distances to every other residue. For each pair of amino acids in the compared protein structures a dynamic programming is used with scoring matrix based on the residue distances (local similarity). In the second-level dynamic programming, the matrices are aggregated to obtain the resulting structure alignment (global similarity).

Another well-known method for comparison of two protein structures is MAMMOTH [9]. MAMMOTH represents each amino acid by its sequence neighborhood that is 7 amino acids long (heptapeptide). The unit-vector root mean square for each pair of heptapeptides is computed and forwarded into the Smith-Waterman algorithm as a scoring matrix. The output of Smith-Waterman forms an alignment of the two structures. Maximum subset of aligned pairs being spatially close after superposition (based on the alignment) is taken into account for computing so-called *percentage of structural identity* (PSI). In the last step, probability of obtaining the given PSI by chance (*P-value*) is calculated as the final result.

More recently, methods based on Voronoi diagrams were proposed. The *Vorometric* method forms contact strings from the Delaunay tessellation and these are stored in a metric index [10]. For finding similar contact string with the query, edit distance with metric scoring matrix is used. The resulting hits are used as seeds for the consequent step, where a modification of dynamic programming is applied to the hits in order to obtain the alignment.

*Vorolign* extracts nearest-neighbor sets for each amino acid based on the Voronoi tessellation [11]. There is a similarity of the sets defined, which is further used in dynamic programming for assessing local similarity to a pair of amino acids. The local similarities are used as scores for second-level dynamic programming. The same group of authors introduced later a solution called *PPM*[12]. PPM identifies sufficiently similar (core) blocks which are then used to create a graph of core blocks. That path in the graph is chosen, that minimizes the cost of mutations.

The *db-iTM* method is a recently proposed solution which represents amino acids as a set of concentric circles [13]. Based on their densities and radii, the method forms feature vectors used in local dynamic programming.

Last of the structure-based methods presented in this overview is *3D-BLAST*[14]. This method derives structural alphabet from the *κ*-*α* plot. The structures represented as strings over this alphabet are accessed using the BLAST approach. That takes us to other methods which are purely sequence-based and thus we are able to provide comparison of structure-based approaches with the sequence-based ones – *BLAST*[15] and *PSI-BLAST*[16]. BLAST is the state-of-the-art tool for similarity search in protein sequence databases. It is based on heuristics which noticeably decreases runtime needed for the full Smith-Waterman algorithm [8], which is the optimal measure for assessing similarity to a pair of protein sequences. PSI-BLAST extends the original BLAST algorithm by employment of a position-specific scoring matrix, so that it is more sensitive to weak sequential similarities.

In this work, we propose our own approach to protein structure similarity, called SProt, based on high-quality modeling of local similarity in the process of feature extraction. SProt’s features are represented by the spherical spatial neighborhoods of amino acids, because on them the similarity can be well-defined. Along with the proposed similarity measure, we also introduce an access method that reduces the number of applications of the measure and so the real-time cost. The effectiveness and efficiency of the proposed approach is evaluated by experiments.

## Methods

In contrast to most of the presented algorithms, in our solution we put a lot of emphasis on high-quality modeling of local similarities of the amino acids. We believe that representing proteins by various derived features might cause loss of information which is inevitable for quality alignment. In this section we present our solution, called *SProt*, which aims to avoid the possible loss-of-information drawback.

### SProt fundamentals

As we have already mentioned, determining alignment and superposition of protein structures is a nontrivial problem. However, what holds true for whole protein structures does not have to be valid for small substructures. If we want to align two small parts of two protein backbones, the natural way is to execute gapless alignment for these parts. When aligning only a few amino acids, it does not make sense to introduce gaps and thus the alignment is defined unambiguously. As stated above, the computation of the superposition of the backbones is then a relatively easy task. We further employ this superposition in a consequent step where we add those amino acids to the alignment that are spatially close to the already aligned backbone amino acids. These do not have to be close in terms of sequence order. In this way, we are able to take the spatial neighborhood into account when modeling local similarity. The above outlined principle is the central point of the local measure used in SProt.

Before we describe the details of the algorithm in the following sections, we briefly present the main ideas. SProt represents each amino acid A by amino acids that are spatially close to A (section *Representation of a protein*). To compute the local similarity between such representations of amino acids, an alignment and superposition are subsequently performed (section *Sphere similarity*), as motivated above. The computed local similarities are then used by a dynamic programing method to obtain the global structural alignment. The quality of this alignment is expressed in terms of a TM-score value (section *Alignment* and *superposition*). The overall computation time can be decreased in the process of querying the database for the most similar protein structure. For this purpose we apply an access method adopted from the field of metric indexing (section *Speedup by indexing*).

### Representation of a protein

Each amino acid A is represented by the amino acids located within the euclidean sphere centered in A and with given radius. Since the representation of A is based on its spatial neighborhood bounded by the sphere, we call the representation an *aa-sphere*.

SProt treats the position of each amino acid as its *α*-carbon position. However, when testing intersection of an amino acid with a sphere, all heavy atoms of the amino acid are considered, not only the *α*-carbon. Such an approach allows us to include amino acids into the aa-sphere whose *α*-carbons are too far from the aa-sphere’s center but their side chains are still close enough.

We divide the content of each aa-sphere into several categories:

• *Spherical backbone* is the maximal continuous part of the amino acid sequence that is included in the aa-sphere and contains the central amino acid. A spherical backbone is divided into *upstream spherical backbone* and *downstream spherical backbone*. In the former the amino acids precede the central amino acid in the protein sequence, while in the latter the amino acids follow the central amino acid.

• *Upstream neighborhood* contains amino acids in the aa-sphere that precede the central amino acid in protein sequence and are not included in the spherical backbone.

• *Downstream neighborhood* contains amino acids in the aa-sphere that follow the central amino acid in protein sequence and are not included in the spherical backbone.

**Figure 1:**
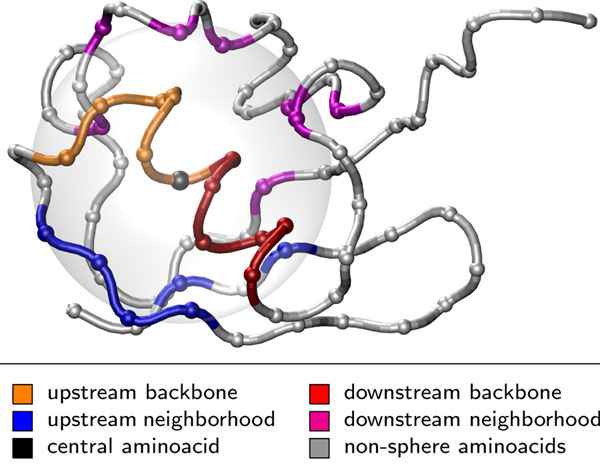
**An example of an aa-sphere** This example demonstrates an aa-sphere for the 26-th amino acid of Ubiquitin [PDB:1UBQ]. Each amino acid is represented by a ball centered in its α-carbon position. The tube corresponds to the protein backbone denoting the protein sequence. The Euclidean sphere with center in the 26-th amino acid (black) with radius 9 Å (gray). The different colors emphasize amino acids included in the aa-sphere. Some of the heavy atoms of the colored amino acids with their *α*-atoms outside the Euclidean sphere intersect with the sphere and thus the respective amino acids are also included in the aa-sphere. The figure has been generated by VMD [30].

See Figure 1 for an example of an aa-sphere, including the categories.

For the purposes of the following steps, the amino acids in each category preserve the original protein sequence ordering. We also define the term *quantity characteristics* for each aa-sphere to denote the number of amino acids belonging to a particular category. The whole protein is then modeled by a sequence of aa-spheres built for every amino acid.

### Sphere similarity

We measure similarity of aa-spheres using alignment and superposition of their content, as this is simpler for aa-spheres than for entire protein structures. Assessing the similarity to a pair of aa-spheres consists of five steps, where the first three steps construct the alignment and the last two valuate it:

1. *Generating seed spherical backbone alignment*. Spherical backbones are aligned using gapless alignment. The alignment is unique since it is gapless and the central amino acids are aligned to each other.

2. *Computing spherical backbone superposition*. The alignment from the previous step determines the spherical superposition carried out by the Kabsch algorithm, which is of linear complexity [2].

3. *Generating spherical alignment*. In the previous step, we have superposed the spherical backbones. However, to assess similarity to the whole aa-spheres, we have to consider also the other aa-sphere content. Therefore, the obtained superposition is used to align the rest of the amino acids in the aa-sphere (upstream and downstream neighborhoods). We apply the Needleman-Wunsch algorithm [17] (global alignment) separately on the upstream and downstream neighborhoods. The algorithm utilizes a scoring function in the form(1)

where *d_ij_* is the euclidean distance of *i*-th and *j*-th amino acids according to the superposition of the aa-spheres, and *d_s_* represents a scale parameter (empirically determined).

4. *Computing raw spherical measure* (*SM-raw*). The raw spherical measure for aa-spheres *x* and *y* is computed for the whole spherical alignment (steps 1, 2, 3) as(2)

where *L_A_* is the length of the alignment, *d_i_* is the distance between *i*-th pair of amino acids according to the spherical superposition, *d_s_* is the same scale parameter as in the previous step, and *max*_[_*_x_*_][_*_y_*_]_ is a normalization factor (the maximal value of the sum that can be obtained for aa-spheres with the same quantity characteristics as the aa-spheres *x*, *y* have).

5. *Computing normalized spherical measure*. An SM-raw value that is expected to occur for a pair of aa-spheres only by chance depends highly on the quantity characteristics of the compared aa-spheres. That is because better superpositions are more probable for smaller aa-spheres. Hence, there arises a problem when comparing the similarities between pairs of aa-spheres with different quantity characteristics. Therefore, we compute the empirical cumulative distribution functions (ECDF) for SM-raw that are specific to quantity characteristics of the compared aa-spheres *x* and *y* (denoted as ). The usage of ECDF allows us to express the probability that a better result could not be obtained by chance for aa-spheres with identical quantity characteristics. However, such a modification is not yet sufficient. For example, if aa-sphere *w* is obtained from aa-sphere *z* by removing some amino acids, then SM-raw(*w*, *z*) will be maximum for given quantity characteristics. It implies that ECDF of SM-raw(*w*, *z*) will be maximal as well, but that is not correct. Therefore, we added the factor *f* that captures the differences in the quantity characteristics of aa-spheres *x* and *y:*(3)

where *q_ub_*, *q_db_*, *q_un_* and *q_dn_* denote individual quantity characteristics of an aa-sphere.

The full normalized measure of the aa-spheres *x* and *y* is then(4)

### Alignment and superposition

To generate the global alignment of two protein structures, the logarithm of SM-score is used as a scoring function together with the linear gap penalty model. The SM-score estimates the probability that a matching of given pairs of spheres is significant. Thus, the logarithm of SM-score used inside the Needleman-Wunsch algorithm maximizes the probability that the resulting alignment is significant. After obtaining the alignment, we employ the widely used TM-score algorithm to get the superposition and the final score [18]. The TM-score algorithm was designed in order to maximize the following formula:(5)

where *L_A_* is length of the alignment, *L_T_* is size of the query structure, d_i_ is distance between *i*-th pair of amino acids according to the superposition computed by the TM-score algorithm, and *d*_0_(*L_T_*) is a scale function.

When speaking about similarity measure, we understand high scores as high similarities. However, for some applications it is more convenient to treat similarity as distance. Thus, similar structures exhibit low distance. Since the TM-score is a similarity measure that reaches 1 for identical structures, it can be easily converted to a distance function as *d*(*x*,* y*) = 1–TM-score(*x*, *y*).

### Optimizations

The proposed SProt similarity measure depends on the following parameters that must be tuned to obtain high-quality results.

#### Sphere radius

This parameter determines the number of amino acids in an aa-sphere. A small radius results in low number of amino acids in an aa-sphere which leads to decreased accuracy. On the other hand, using a large radius increases the time needed to compute the aa-sphere similarity. This is because a large aa-sphere influences the runtime of the Needleman-Wunsch algorithm (being of quadratic complexity).

In our experimental section, we used sphere radius 9 Å as a trade-off between time and accuracy.

#### Scale parameter d_s_

The SM-raw measure is a variant of TM-score that uses scale parameter dependent on the size of the compared proteins. However, TM-score’s parameterization is not suitable for aa-spheres, because they are much smaller than the whole protein structures. Therefore, we used constant-value scale parameter as the ancestors of TM-score did. For example, MaxSub [19] used value 3.5 Å, S-score [20] used value 5 Å. We decided to set the parameter to 2 Å due to the generally smaller sizes of aa-spheres in comparison to the average protein size.

#### SM-raw empirical cumulative distribution functions

The empirical cumulative distribution functions (ECDF) of SM-raw measure were produced from the all-to-all comparisons of proteins taken from ASTRAL-25 v1.65 database [21]. Since the ECDF computation is highly space-consuming if every possible combination of quantity characteristics has to be taken into account, a downsampling technique was used to decrease the space complexity. The upstream and downstream neighborhood characteristics were downsampled by a factor of 2, the backbones of sizes 0 and 1 were treated identically as well as each quantity characteristics exceeding value 7.

#### Gap penalty

Setting a gap penalty value has the essential impact on the quality of the measure. We used *log*(0.75) as the gap penalty value which has the best results for most of the evaluations. This setting of the gap penalty is low enough, thus only amino acids with significant similarity will be paired.

### Speedup by indexing

The proposed SProt measure is computationally very expensive. This poses a challenge especially in the task of selecting the most similar structures from a large structure database where many SProt computations have to be performed. One of the possible solutions of this challenge is to employ indexing methods.

#### Metric access methods

Most of the domain-specific applications of similarity search employ pairwise similarity only as a step within the process of database search. Typically, we search for the most similar object in a database to a given query. The most straightforward solution in such a scenario is to sequentially scan the database, compare the query object to each object in the database and identify the most similar object (the nearest neighbor) or the *k* most similar objects (the *k* nearest neighbors).

The *metric access methods* (or metric indexes) [22] form a set of index structures allowing to filter out database objects not similar to the query, thus highly decreasing the runtime while maintaining accuracy of the search. The goal is achieved by resorting to metric distance functions, which is the requirement of all metric access methods. Hence, only the domains where the distance *d* between objects fulfills the metric axioms can benefit from the metric access methods (without loss of accuracy). The metric axioms are as follows (∀*x*, *y*, *z*):

1. *Non-negativity*: *d*(*x*, *y*) ≧ 0

2. *Identity of indiscernibles*: *d*(*x*, *y*) = 0 iff *x* = *y*

3. *Symmetry*: *d*(*x*, *y*) = *d*(*y*, *x*)

4. *Triangle inequality*: *d*(*x*, *z*) ≦ *d*(*x*, *y*) + *d*(*y*, *z*)

The axiom of triangle inequality is the most important for metric access methods. This axiom, in conjunction with the other ones, allows to compute a lower bound *d_LB_*(*q*, *o*) of the distance *d*(*q*, *o*) between a query object *q* and a database object *o* through another database object *p* (often called a pivot). Specifically, the following equation follows directly from the axioms:(6)

It is possible to compute multiple lower bounds of the distance by using different pivots and select the maximum lower bound being the closest one to the distance. This can provide a good estimate of the distance between *q* and *o*. If the estimate is large enough, object *o* can be filtered out, because it surely cannot be close to the query and so cannot be a part of the result set.

One of the metric access methods, representing so-called pivot-based approach, is LAESA [23,24] (Linear Approximating and Eliminating Search Algorithm), being suitable for time-expensive measures because of its filtration abilities [25]. LAESA uses a small part of the database as the set of pivots. The pivots are used during the query process to estimate distances between a query and all the database objects. Based on these estimates, it is possible to eliminate some of the database objects from the search, so that the expensive distance computations between the query and these objects are not needed to compute. To compute the distance estimations as fast as possible, all distances between the pivots and the database objects are precomputed and stored in so-called *metric index*.

To perform the *k* nearest neighbor query, LAESA maintains a set *S* containing not yet eliminated objects that might be still included in the result. The elimination process is based on estimations of distances between the query and database objects. Thus, LAESA also maintains the estimation of the distance for the query and each database object *o* (*e*(*o*)). These estimations are continuously updated as more and more pivots are taken into account. During the execution of the algorithm, the *k* nearest neighbors from the set of already processed objects are stored in a set *R*. At the end of the algorithm, the set *R* contains the final result, i.e., the *k* nearest neighbor objects.

The LAESA algorithm can be described as follows:

1. *Initialization*: At the beginning, all database objects might be included in the result, therefore the set *S* contains all database objects. Lower-bound estimations of distances between the query and database objects are set to 0 and the set *R* is empty.

2. *The first pivot selection*: An arbitrary pivot is selected and denoted *s*.

3. *The main loop*: While *s* is defined:

(a) *Distance computation*: Remove *s* from the set *S* and compute distance *d*(*q*,*s*). Update the set *R* to contain *k* already processed objects having the smallest distances to the query object *q*.

(b) *Approximation:* If *s* is a pivot, use it to make the estimations more accurate. That is, for each database object *o*, compute a lower bound of its distance to the query and set the related estimation *e*(*o*) to the value of the lower bound if the lower bound is greater than the original value of the estimation.

(c) *Elimination*: Use the greatest distance between the query and an object from *R* as a threshold and eliminate all objects *o* from *S* having *e*(*o*) greater than the threshold. The distance between *o* and query *q* is never smaller than the related estimation *e*(*o*), thus the eliminated objects cannot be included in the result. However, pivots contained in the set *S* are explicitly protected against elimination during the first few steps. The number of such steps is a parameter of the algorithm.

(d) *The next object selection*: If *S* contains pivots, select a pivot *p* ∈ *S* having the smallest estimation *e*(*p*) and denote it as *s*. Otherwise, select *b* ∈ *S* having the smallest estimation *e*(*b*) and denote it as *s*. If *S* is empty, *s* becomes undefined and so the algorithm terminates.

4. *Result*: The set *R* contains results of the search.

#### Capability of indexing

From the description of LAESA (step 3c) it follows that the speed-up is directly proportional to the number of objects eliminated during the query process. It has been shown [26] that the elimination ability (indexability) depends of the distribution of the distances between objects in the metric space. If a distance exhibits low degree of indexability, it could be improved by applying a convex function on top of the original distance, the so-called *similarity-preserving modifier*[*26*]. The modifier virtually makes the object clusters in the database more tight, so that the indexability is increased. However, the use of such a modifier may violate the triangle inequality axiom to some extent. In particular, for some triplets of the database objects *x*, *y*, *z* the triangle inequality formula does not hold, which can cause inaccuracies in the search. In such case the search becomes only approximate. Therefore, the modifier has to be chosen carefully since it represents the trade-off between accuracy and speed.

#### SProt access method

In contrast to what has been stated above, unfortunately, SProt is not a metric distance, because it does not satisfy symmetry and triangle inequality. The absence of symmetry does not form a serious problem — a small change in the lower bound formula 6 can fix it:(7)

It is important to note that this formula requires (due to asymmetry) to compute both of the distances *d*(*q*, *p*) and *d*(*p*, *q*). Both computations share the same alignment, utilizing more than 90% of the computation time. Hence, *d*(*p*, *q*) can be computed relatively cheap when *d*(*q*,* p*) is already computed. A more substantial problem is that SProt violates the triangle inequality, although the number of the violating object triplets is small. However, it is important to realize that even a relatively small probability that a triplet violates the axiom can lead to a high probability that an estimation produced by LAESA during the execution is overvalued and so is incorrect. For example, suppose that the probability that a triplet does not satisfy the axiom is 10^–4^. Then, if we used 1000 pivots to estimate a distance, the probability that the estimation is incorrect would be approximately 1 – (1 – 10^–4^)^1000^ ≈ 9.5%. The reason is that the estimation of a distance is always set to the maximum of lower bounds produced by different pivots. Thus, if one of the lower bounds is overvalued, then the estimation is overvalued as well, so that the estimate becomes incorrect. 

Therefore, it is desirable to adjust the method to be more robust against incorrect estimations. To do so, we introduced two enhancements:

1. An object *t* is eliminated during the LAESA elimination step if the estimation *e*(*t*) of *d*(*q*, *t*) is greater than a threshold *θ*. In such case the distance *d*(*q*, *t*) is greater than *θ*. However, it may not be true if the estimation is overvalued. Hence, we introduced requirement that the estimation must be greater than *θ* by more than *v* percent to make the algorithm robust against small overvaluation in the estimations. If the estimations are not overvalued by more than *v* percent, then the result of the algorithm is equal to sequential scan. We call the *v* value the *approximation error tolerance factor*.

2. The second improvement does not depend directly on the rate of overvaluation. Assume that *s* is included in the result corresponding to the sequential scan. Then, if the algorithm processes *s* in the main loop, *s* has to be added into the set *R* and will be never pushed away by any other object. This is because there are no more than k – 1 objects in the database having smaller distances to the query than *s* has. Thus, all incorrectness in the result can be interpreted as a too early elimination of the object (due to its overvalued estimation) before it could be processed.

   Once all objects are eliminated, the main loop is terminated. Hence, the second improvement is intended to delay the termination of the main loop and to process some of the eliminated objects. Originally, the main loop is terminated after there is no object *s* to be selected from *S*. Thus, we modify the step of the next object selection. If the set *S* is empty, the eliminated objects are taken into account and an eliminated object *b* with the smallest estimation *e*(*b*) is selected and denoted as *s*. This type of selection can be performed up to *r* times since the last change of the set *R*. In other words, once the original stop condition is true the stability of the set *R* must be additionally confirmed by *r* consecutive iterations of the main loop during which *R* must not be changed. We call the *r* value the *order error tolerance factor*. This factor makes the method more robust against some incorrectness caused be wrong order of objects’ selection due to incorrect estimations.

Proper settings of the introduced factors will prevent from incorrect estimations. As we show later, this prevention is so good that the use of modifiers improving indexability is possible. However, it is important to note that the searching is still approximative.

## Results

In this section, we evaluate SProt from two points of view. First, we assess the quality of SProt in terms of retrieval effectiveness. Second, we examine the efficiency (speedup) of search using SProt.

### Effectiveness

In order to evaluate the quality of the proposed measure, we focus on expressing how well the measure fits the view of experts on protein structure similarity. The difficulty of this task lies in the absence of a large-scale expert-moderated database of pairwise protein structure similarities, which we could use as a standard of truth. However, there exists the expert-moderated hierarchical evolutionary classification SCOP (structural classification of proteins) that could be used for this purpose [27]. Using SCOP, we are able to (indirectly) compare SProt with domain expert’s conception of the structure similarity. The SCOP hierarchy consists of four levels – *family*, *superfamily*, *fold* and *class*. Proteins in the same family have either high sequence similarity (> 30 %), or they have a lower sequence similarity (> 15 %) but share very similar function or structure. Proteins that share common evolutionary origin (based on structural and functional features) but have different sequence reside in the same superfamily. Structures that share major secondary structures in similar topological distribution are in the same fold. And finally, similar folds are grouped into classes.

Therefore, SCOP can provide us with the information whether two protein structures are considered similar or not (at the given level) by a human observer. Although such a binary measure (similar or dissimilar) is not able to express detailed qualities of the similarity measure, such as the quality of alignment or superposition, it is suitable to express performance of the measure in terms of ability of classification and retrieval.

#### Protein classification

Automatic classification of protein structures is one of the traditional problems. The task is to determine SCOP classification of a query protein according to the investigated measure. The category of the query protein is derived from category of the database protein being most similar to the query. Accuracy of classification at a given level is measured as the percentage of correctly classified queries.

We used the dataset that was originally introduced for evaluation of the *Vorolign* method (*Vorolign dataset*). The dataset utilizes ASTRAL-25 v1.65 [21] containing 4,357 structures. As the query set, 979 structures from difference set between SCOP v1.67 and v1.65 are used.

Results on this datasets are summarized in Table 1. The table describes the classification accuracy for family, superfamily and fold levels. It also shows average values of several characteristics describing the algorithms from different points of view. Namely, the table contains average TM-score, average RMSD and average alignment cover (i.e., how many percent of amino acids of a query is aligned) between each query and its most similar structure used for classification. At the superfamily and fold level, SProt outperforms the other solutions, while at the family level SProt is slightly defeated by Vorometric. It is interesting to realize that although the other solutions stand out in terms of average values of the various characteristics, SProt outperforms them in terms of classification accuracy. Thus, better partial characteristics do not necessarily lead to better real-world results.

**Table 1:** Classification accuracy

Method	Family	Superfamily	Fold	RMSD	Cover	TM-score
SProt	90.4	**96.9**	**98.6**	4.14	81.1	0.63
Vorometric	**90.7**	94.9	97.6	2.43	**87.2**	0.74
PPM	88.3	94.5	97.5	n/a	n/a	n/a
db-iTM	86.6	95.8	98.2	n/a	n/a	n/a
Vorolign	86.4	92.4	97.7	**1.90**	76.3	0.74
CE	84.6	91.9	94.1	1.95	78.2	**0.77**
BLAST	48.9	52.5	52.8	–	–	–

The evaluation of classification accuracy was performed on the Vorolign dataset for different levels of the SCOP hierarchy (family, superfamily and fold). The table also describes average characteristics (RMSD, alignment cover and TM-score) of the most similar structures to the query. The values of the db-iTM method are taken from [13], and the values of the other compared methods are taken from [10].

#### Information retrieval in protein structure databases

In the previous section, we measured the hit rate based on the most similar database structure. Thus, the most similar structure was the only determinant of the quality. However, the user often wants to obtain *all* relevant structures, not just the most similar one. The result can be then visualized as a list of database structures ordered according to the given measure with the most similar structure on top. Correctness of such ordering can be measured in terms of *precision* and *recall*, used as standard effectiveness measure in the area of information retrieval [28]. Precision expresses how many percent of structures at the given cut-off rank in the result list are relevant. Recall expresses how many percent of all relevant results are obtained at the given cut-off rank in the result list. The precision-recall dependence can be expressed in a graph that describes the average precision of queries for different recall levels. As a single-value evaluation metric, it is possible to use the widely accepted *mean average precision*[28]. For a single query, the *average precision* is defined as the average of precision values that are computed for prefixes in the result list, where each of the list prefixes ends by a relevant structure. The mean of these values for all queries then determines the mean average precision.

Another single-value evaluation metric is described in the Vorometric paper [10] and called here also *average precision*. To avoid a confusion we will call it *average precision for standard recall levels*. This evaluation metric is defined as the mean of average precision values for the 10 standard recall levels (10%–100%).

For this experiment we used the *ProtDex2 dataset* consisting of 34,055 proteins that have been originally used for evaluation of the ProtDex2 method. As the query set, 108 structures from medium-size families of the dataset were selected.

**Figure 2:**
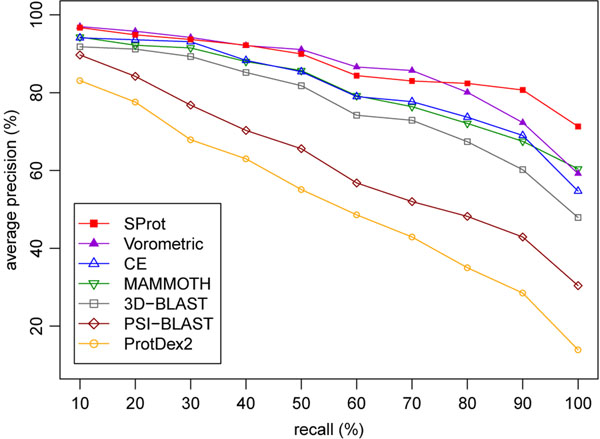
**Average precision-recall curves** The curves were computed for all 108 queries of the ProtDex2 dataset. The data for the compared methods are borrowed from [10].

We consider a selected database structure as relevant if it comes from the same SCOP family as the query. Precision-recall graph for the used dataset is presented in Figure 2. The SProt has better precision-recall curve than the other methods, except Vorometric. In comparison with Vorometric, the curve of SProt is slightly worse for medium recall levels while it is noticeably better for high levels. When measuring the above defined single-value evaluation metrics, SProt outperforms the other methods as Table 2 demonstrates.

**Table 2:** Average precision

Method	Mean average precision	Average precision for standard recall levels
SProt	**88.3**	**86.9**
Vorometric	n/a	82.9^1^
CE	83.4	80.9
MAMMOTH	82.1	80.8
3D-BLAST	78.2	76.2
PSI-BLAST	69.8	61.8

The experiments was performed against the ProtDex2 dataset and all 108 queries were utilized. The mean average precision values of the compared methods are taken from [14], and the average precision for standard recall levels values of the compared methods are taken from [10].

^1^ based on returning top 100 hits

#### Quality of structural alignments

It would also be appropriate to investigate what is the quality of alignments and scores the measure produces. For this purpose, 10 difficult pairs of structures were introduced in [29]. It is obvious from Table 1 that SProt does not produce high alignment cover and TM-score. However, to produce better alignment and TM-score, it is possible to apply iterative improvement of TM-score. In this case, the superposition obtained by the original SProt is used to produce a new better alignment. A similar approach was utilized also by other methods, e.g., Vorometric. For the purpose of the improvement, Needleman-Wunsch algorithm is used with the scoring function(8)

where *d_ij_* represents distance between *i*-th and *j*-th amino acid according to the superposition, *L_T_* is the length of the query protein, and *d*_0_(*L_T_*) is the scale function used in TM-score. The 3*d*_0_(*L_T_* ) threshold is used to prevent aligning too distant amino acids. The resulting alignment is then used in the TM-score algorithm to obtain new score and superposition. This procedure is repeated while the score is being improved.

As shown in Table 3, this approach (denoted SProt + TM-optimization) significantly improves the cover and score. On the other hand, extensive use of the iterative concept does not improve the results of the previous evaluations whereas it noticeably downgrades performance of the algorithm.

**Table 3:** Comparison of the alignment quality

Method	RMSD	Cover	TM-score
SProt + TM-optimization	3.29	85.8	**0.65**
SProt	7.29	73.8	0.43
Vorometric	3.02	84.8	**0.65**
Vorolign	**2.28**	51.7	0.56
DaliLite	2.82	80.0	0.61
SSAP	4.37	**88.1**	0.59
CE	3.17	83.4	0.60

The tests were performed on the special set of 10 difficult pairs of structures and average values of various characteristics are presented. The values of the compared methods are taken from [10].

### Efficiency

To evaluate the speedup possibilities based on indexing, we utilized the ProtDex2 dataset. This dataset is large enough to show advantages of indexing. On the other hand, it is still size that allows to perform the sequence scan in a reasonable time. Hence results of sequence scan can be used for comparison.

The following settings were used. We selected 1651 protein structures as pivots, one from each family in the dataset. The value tolerance factor was set to 2.5% and the order tolerance factor was set to 128. The number of steps over which the pivots were protected against elimination was set to  of the total number of pivots. These settings provide sufficient robustness to prevent overvalued estimations for the used dataset.

**Figure 3:**
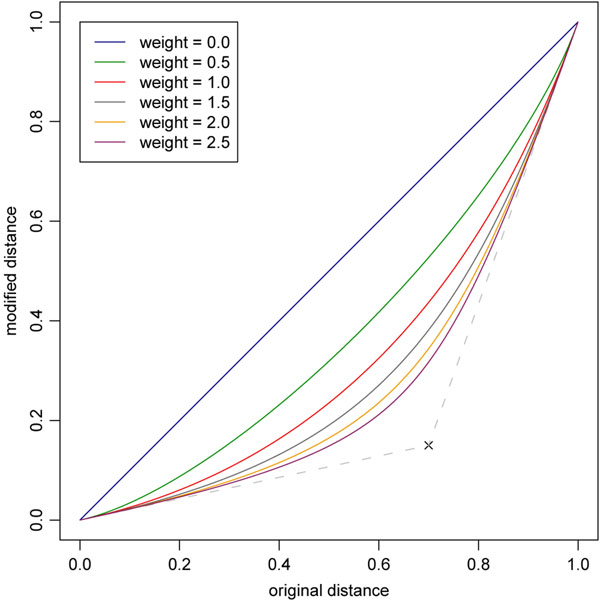
**RBQ modifiers** RBQ_(*a*,*b*)_(*w*) modifier is defined as the rational Bézier quadric curve starting at point [0, 0] going toward the control point [*a*,* b*] ([0.7, 0.15] in this case) and arriving to point [1, 1]. Weight *w* determines the degree of deflection of the curve toward the control point.

A more important property is the impact of the employed modifiers that seamlessly balance between the retrieval accuracy and the speed. The SProt measure ranges from 0 to 1 while most of the distances (approximately 95%) are higher than 0.7. Therefore, we decided on the basis of our experience to use a modifier that, simply said, smoothly expands the interval [0.7:1] at the expense of the interval [0:0.7] which is condensed. One of such modifiers is the *RBQ*_(0.7,0.15)_(*w*) modifier [26] parametrized by a weight *w*. This modifier is defined as the rational Bézier quadric curve, starting at point [0, 0] going toward point [0.7, 0.15] and arriving in point [1, 1]. The weight *w* determines the degree of deflection of the curve toward the point [0.7, 0.15] (i.e., the convexity of the function). Thus, the weight *w* determines the ratio of the expansion and condensation and thus it also impacts the indexability. The *RBQ*_[0.7,0.15]_(*w*) modifier for various weights *w* is depicted in the Figure 3.

**Figure 4:**
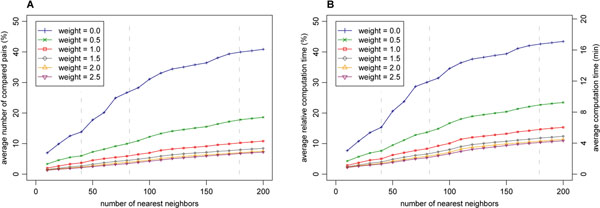
**Access method efficiency** The efficiency of the SProt access method was measured for different weights of the modifier and for different numbers of requested nearest neighbors. All the 108 queries of the ProtDex2 dataset were utilized and the average values are presented. The efficiency is expressed both in terms of the relative (according to sequential scan) number of protein structure pairs being compared (A) and in terms of the relative (according to sequential scan) computation time (B). Figure (B) also includes the absolute time which was measured on a machine containing an Intel Xeon E7540 2.00GHz processor. The sequential scan takes 39.4 minutes on average. Vertical dashed lines denote minimal, average and maximal size of the query families in the dataset.

As shown in Figure 4, the computation time and the number of protein structure pairs being compared increases with the decreasing weight, and they also naturally increase with the increasing number of the requested nearest neighbors.

It is also important to describe the precision of such approximative searches. The precision of approximate search using *k*-nearest neighbor query is measured as the *retrieval error* between the query result returned by the SProt access method (*R*(*q*)) and the accurate query result obtained by sequential scan of the database (*R_seq_*(*q*)):(9)

The retrieval error describes how many percent of structures included in the sequential scan result are missing in the result of the SProt access method.

**Figure 5:**
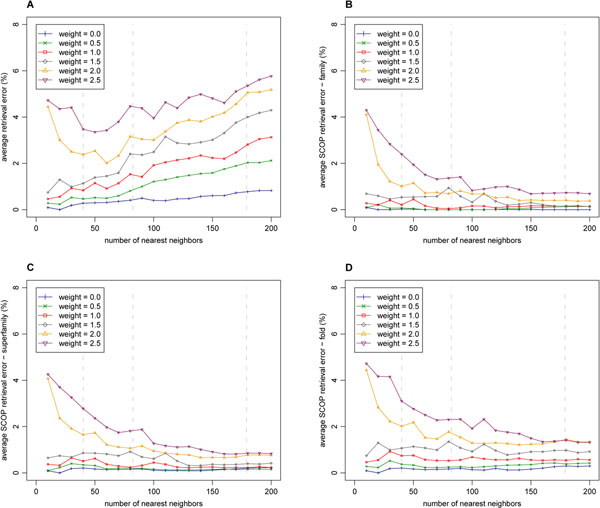
**Retrieval errors** The retrieval errors of the SProt access method were measured for different weights of the modifier and for different numbers of requested nearest neighbors. All the 108 queries of the ProtDex2 dataset were utilized and the average values are presented. The errors are described as the percentage of protein structures included in the sequential scan result and missing in the result of the SProt access method. In the first case (A), the whole result of sequence scan is considered and the retrieval error E is measured. In the other cases, only subsets of the result sharing the same SCOP family (B), superfamily (C) or fold (D) with the query structure are taken into account, leading to different SCOP retrieval errors. An error 0% means that none of the considered structures were missing, and conversely, an error 100% means that all considered structures were missing. Vertical dashed lines denote minimal, average and maximal size of query families in the dataset.

As shown in Figure 5A, the retrieval error naturally increases with the increasing weight. Moreover, with the increasing number of the requested nearest neighbors, the error increases. An exception is the retrieval error for high weights and low numbers of the requested nearest neighbors, where it also increases. However, with the increasing number of the requested nearest neighbors, the retrieval error becomes less significant. The reason is that missing structures are often located in the back positions of the result. As shown in the information retrieval experiment, at the back positions there are located relatively few of the relevant structures (according to meaning of domain experts). So, we also introduce SCOP retrieval errors that takes into account the SCOP categories (family, superfamily or fold):(10)

where *R*(*q*) is the result set obtained by the method for query *q* and *R_seq_*(*q*) is the sequential scan result set and *S_L_*(*q*) is a set of all structures having the same SCOP category at level *L* (at the family, superfamily or fold level) as the query structure *q*. Thus, the SCOP errors describe how many percent of relevant structures included in the sequential scan result are missing in the result of the SProt access method. As it can be seen in Figure 5, the SCOP errors still naturally increase with the increasing weight. Nevertheless, the errors do not negatively depend on the number of the requested nearest neighbors and they are very small. Again, the exceptions are the errors for high weights and low numbers of the requested nearest neighbors.

When searching in ProtDex2 dataset, we could conclude that the weight value of the modifier set close to 1 results in reasonably fast retrieval and low retrieval errors. However, the optimal configuration of the SProt access methods parameters may vary, depending on the dataset used (especially on its size and indexability). However, it is important that the above factors (except from pivot selection) do not need to be know during the database indexing and can be set right at the query time. Thus, the user has the freedom to change the settings if he is not satisfied with the obtained results, and run the query again using the same index.

## Conclusions

In this paper, we proposed SProt – a novel algorithm for measuring protein structure similarity that puts emphasis on high-quality modeling of local similarities of the amino acids. This is achieved by representing each amino acid by its spatial neighborhood containing close amino acids. The approach leads to good real-world results, especially for superfamily/fold classification accuracy and for precision at high recall levels where we outperform all the compared solutions. The focus on the quality of the modeling results in high computational demands of the method. We resolve this handicap be introduction of SProt access method – a modification of LAESA metric access method – that highly decreases the runtime needed for scanning large datasets of protein structures. The speedup makes SProt competitive with the best contemporary solutions not only concerning the effectiveness but also the efficiency.

## Abbreviations

ECDF: empirical cumulative distribution function; LAESA: linear approximating and eliminating search algorithm; NP-hard: non-deterministic polynomial-time hard; PSI: percentage of structural identity; RBQ: rational Bézier quadratic; RMSD: root mean square deviation; SCOP: structural classification of proteins;

## Competing interests

The authors declare that they have no competing interests.

## Authors contributions

JG designed and implemented both the measure and the access method. DH critically revised the both. JG performed all the measurements. JG and DH drafted the manuscript. TS critically revised the manuscript and gave final approval of the work to be published. All authors read and approved the final manuscript.
